# Small molecule immunomodulation: the tumor microenvironment and overcoming immune escape

**DOI:** 10.1186/s40425-019-0667-0

**Published:** 2019-08-22

**Authors:** Arsen Osipov, May Tun Saung, Lei Zheng, Adrian G. Murphy

**Affiliations:** 1 Department of Oncology, The Sidney Kimmel Comprehensive Cancer Center, Johns Hopkins University School of Medicine, Baltimore, MD USA; 2 GI Oncology, Sidney Kimmel Comprehensive Cancer Center, Harry and Jeanette Weinberg Building, CRB1 1, Room 487, 1650 Orleans Street, Baltimore, MD 21231 USA

**Keywords:** Small molecules, Immunomodulation, Immunotherapy, Focal adhesion kinase, Tumor microenvironment, Colony stimulating factor, Immune escape

## Abstract

Immunotherapy has led to a paradigm shift in the treatment of many advanced malignancies. Despite the success in treatment of tumors like non-small cell lung cancer (NSCLC) and melanoma, checkpoint inhibition-based immunotherapy has limitations. Many tumors, such as pancreatic cancer, are less responsive to checkpoint inhibitors, where patients tend to have a limited duration of benefit and where clinical responses are more robust in patients who are positive for predictive biomarkers. One of the critical factors that influence the efficacy of immunotherapy is the tumor microenvironment (TME), which contains a heterogeneous composition of immunosuppressive cells. Myeloid-derived suppressor cells (MDSCs) and tumor-associated macrophages (TAMs) alter the immune landscape of the TME and serve as facilitators of tumor proliferation, metastatic growth and immunotherapy resistance. Small molecule inhibitors that target these components of the TME have been developed. This special issue review focuses on two promising classes of immunomodulatory small molecule inhibitors: colony stimulating factor-1 receptor (CSF-1R) and focal adhesion kinase (FAK). Small molecule inhibitors of CSF-1R reprogram the TME and TAMs, and lead to enhanced T-cell-mediated tumor eradication. FAK small molecule inhibitors decrease the infiltration MDSCs, TAMs and regulatory T-cells. Additionally, FAK inhibitors are implicated as modulators of stromal density and cancer stem cells, leading to a TME more conducive to an anti-tumor immune response. Immunomodulatory small molecule inhibitors present a unique opportunity to attenuate immune escape of tumors and potentiate the effectiveness of immunotherapy and traditional cytotoxic therapy.

## Introduction

The emergence of immunotherapy has created a paradigm shift in the approach to treating cancer. By leveraging and stimulating the immune system, immunotherapy provides a new avenue to combat advanced cancers. The backbone of treatment for most solid malignancies has traditionally involved cytotoxic chemotherapy. Yet, this modality is associated with significant adverse toxicities and has limitations in providing sustained clinical responses or long-term remissions. These limitations led to the investigation of novel strategies in an attempt to circumnavigate traditional cytotoxic therapy. In 1996, Leach et al., proposed that the inhibition of immune checkpoint cytotoxic T-lymphocyte associated protein-4 (CTLA-4) may lead to an effective anti-tumor response by suppressing the down-modulation of T-cell activation within the immune system and tumor environment [1]. Nearly 15 years later, a seminal clinical study demonstrated that antibody-mediated inhibition of CTLA-4 led to a significant improvement in overall survival in patients with advanced melanoma [2]. These patients, until that moment, had advanced treatment-refractory disease with limited therapeutic options. However, CTLA-4-targeted therapy permanently altered the landscape for the treatment of melanoma, as well as several other aggressive malignancies. These events marshalled the first FDA approval for checkpoint inhibitor immunotherapy with ipilimumab (Yervoy®). Since then, there has been a renaissance with immunotherapy-based treatments for many advanced malignancies. Antibodies targeting other immune checkpoints, such as programmed cell death-1 (PD-1) and its ligand (PD-L1), now have multiple approvals in advanced oncologic indications, such as non-small cell lung cancer (NSCLC), microsatellite-instable colorectal cancer (CRC), renal cell carcinoma, head and neck squamous cell cancer, classical Hodgkin lymphoma, primary mediastinal large B-cell lymphoma, urothelial carcinoma, gastric cancer, cervical cancer, hepatocellular carcinoma (HCC), merkel cell carcinoma, as well as FDA’s first tissue/site-agnostic approval for advanced solid tumors that are microsatellite instability-high (MSI-H) or mismatch repair deficient (dMMR) [3–6].

Despite the ongoing revolution with immune checkpoint inhibition and the success appreciated across many tumor types, more studies have also recognized the limitations of immunotherapy. Several types of malignancies, such as pancreatic cancer are less responsive to immunotherapy than “hot tumors” such as melanoma or NSCLC, which have enjoyed relatively spectacular responses with checkpoint blockade-based monotherapy [7–11]. Even in malignancies where checkpoint inhibitors have received regulatory approvals, the responses are limited to a small subset of patients and tend to be more pronounced in those who are positive for predictive biomarkers. Moreover, there is significant heterogeneity with regard to degree of treatment responses and duration of benefit among various histologies of cancer. Data from current studies suggest that the response to checkpoint inhibition via anti-CTLA-4, PD-1 and PD-L1 is around 15–20% across different tumor types [12–14].

**Fig. 1:**
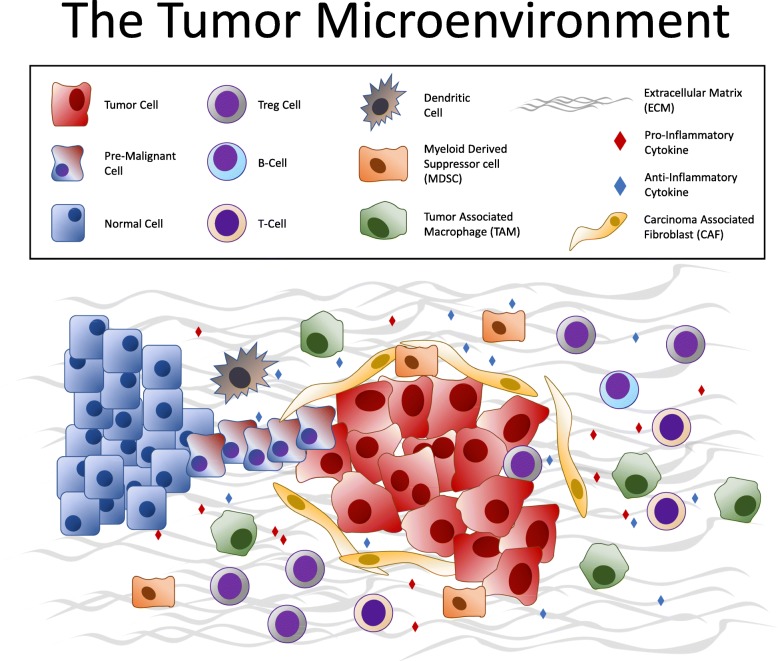
Major cellular constituents and mediators of the TME, including cancer cells, immune cells (T-cells, B-cells, dendritic cells, MDSCs, TAMs), cytokines, CAFs and the extracellular matrix

Much of contemporary research is now focused on understanding the immunosuppressive biology of tumors that leads to immune escape in non-immunogenic or “cold” tumor types and the role the tumor microenvironment (TME) plays in limiting the effectiveness of immunotherapy. The TME is an important facilitator of immune escape and cancer progression [15]. The interaction of malignant cancer cells and the heterogeneous cells within the TME are critical to carcinogenesis. The TME contains cancer cells, immune cells [T-cells, B-cells, dendritic cells, myeloid-derived suppressor cells (MDSCs), tumor-associated macrophages (TAMs)], carcinoma-associated fibroblasts (CAFs), tumor vasculature and lymphatics, as well as adipocytes. Beneath the backdrop of these cells and within a mesh of collagen and elastin fibers that comprise the extracellular matrix (ECM), exists a vast, complicated and constantly changing system of cytokines, growth factors and matrix remodeling enzymes [16]. As a whole, a cancerous mass is composed of as much TME-related nonmalignant cells as it is of purely clonal cancerous malignant cells. Whether it is the immunosuppressive cells or the structural components of the ECM that promote therapy resistance, the TME is a chief mediator of tumor progression and therapy resistance (Fig. 1).

The combination of immune checkpoint monoclonal antibodies (mABs) has been employed to increase the rate of response in “cold” tumors, but these combinations come with an increase in the rate of intolerable toxicities [17, 18]. Immunomodulatory small molecule inhibitors in combination with immune checkpoint mABs, however, have been reported to be well-tolerated in clinical trials [19]. Their smaller size also allows for deeper tissue penetration, and they have the advantage of easier dose administration than mABs, as most small molecule inhibitors are often administered orally [20]. Like mABs, immunomodulatory small molecule inhibitors are also being investigated as monotherapies or as adjunctive therapies to other immunotherapies, targeted therapies or cytotoxic chemotherapy.

Within the TME, there is a complex interplay between mediators of anti-tumor immunity and immunosuppression, ever changing the balance between tumor growth and tumor eradication. There are ongoing preclinical and clinical investigations of small molecule inhibitors that modulate the pathways, barriers and mediators involved in the TME and the immune escape of cancers. Two attractive targets for inhibition are colony stimulating factor-1 receptor (CSF-1R) and focal adhesion kinase (FAK), given their important and intertwined roles in regulating the survival and migration of TAMs – cells which have consistently been associated with tumor progression and poor prognosis [21]. CSF-1R is a receptor tyrosine receptor that is an important regulator of myeloid cell differentiation, proliferation, migration and survival, and FAK is a non-receptor tyrosine kinase that is a critical regulator for macrophage migration [22]. Not surprisingly, high expression of CSF-1R or its ligand, CSF-1, in cancer, including pancreatic ductal adenocarcinoma (PDAC), is associated with poor prognosis and an immunosuppressive TME [23–25]. Presence of phosphorylated FAK (p-FAK) has also been associated with cancer invasion and poor prognosis in numerous cancers [26].

In this review, we will focus on small molecule inhibitors of CSF-1R and FAK, and their therapeutic potential as anti-tumor agents and immunomodulators within the TME.

## Tumor immune microenvironment

The milieu of cells within the TME often serve as a barrier to immune activity and is one of the critical reasons why immunotherapy may have limited clinical efficacy in certain “cold” malignancies like breast and pancreatic cancer [27–29]. The work in the past few decades have come to support and elaborate upon Virchow’s observation in 1863 of leukocytic infiltration within tumor specimens and his pioneering hypothesis that chronic inflammation has significant implications on tumor growth and survival [30].

### Myeloid cells

In addition to T-cells, the vast majority of tumor-associated leukocytes within the TME are myeloid cells, predominantly MDSCs, macrophages and neutrophils in varying stages of differentiation [31]. Subsets of these myeloid cells have been shown to promote, carcinogenesis, angiogenesis and metastasis [32]. MDSCs and TAMs are the two primary myeloid culprits that facilitate the immunosuppressive nature of the TME. Although both are derived from a common myeloid progenitor, there is significant heterogeneity among the myeloid cell populations of cancer, and it is now thought that myeloid cells in tumors exist within a spectrum of differentiation from monocytes/M-MDSCs towards TAMs [33]. MDSCs are classified as polymorphonuclear (PMN)-MDSC or monocytic (M)-MDSC, reflecting their similarities to neutrophils and monocytes, respectively. Current studies have shown that in general, M-MDSCs and PMN-MDSC are explicitly tumor-promoting, whereas TAM are duplicitous in their nature, exerting both anti- and pro-tumor effects [34, 35]. Not surprisingly, the presence and penetration of these cells within the tumor tissue are associated with poor prognosis [36, 37].

Understanding macrophage phenotype polarization is important to elucidating their role in malignancy. Within any tissue, particularly in tumors, macrophage activation can proceed along two vastly different macrophage phenotypes; where the “M1” phenotype is considered pro-inflammatory and “M2” is considered anti-inflammatory [38]. Phenotypic expression of macrophages is dependent on signals from their microenvironment, such as cytokine expression. In healthy tissue, macrophages exist in equilibrium between M1 and M2 phenotypes. However, in progressive cancers, the phenotype is driven towards M2 and skewed away from an M1 phenotype, and M1 phenotype has been noted in regressing tumors [39–41]. In pancreatic cancer, anti-inflammatory pro-tumor polarized macrophages, are associated with increased invasiveness secondary to elevated lymphatic vessel density and significantly poor prognosis [42].

### Cytokines

Within the TME, TAMs and MDSCs are in a background of cytokines that lead to chronic inflammation as well as immune evasion. Inflammatory cytokines, such as tumor-necrosis factor-α (TNF-α), interleukin-6 (IL-6) and IL-8, are often upregulated and promote the invasive properties of cancer, such as angiogenesis and metastasis [43, 44]. Other cytokines, such as IL-4, IL-13 and IL-10, have been reported to be propagators of an anti-inflammatory environment and facilitators of adaptive immune response suppression [41]. Together, the chronic inflammatory milieu and the purveyors of immune evasion modulate the TAM and MDSCs towards promoting tumor proliferation, therapy resistance and metastatic growth [45, 46]. There is also significant crosstalk between MDSCs and other immunosuppressive cells, such as regulatory T-cells (Tregs), which further promotes immune silencing within the TME via cytotoxic CD8+ T-cell inactivation and anergy [47].

In multiple xenograft models, cytokines such as CSF-1 are not only attractants of myeloid cells, like MDSCs and TAMs, but also as promoters of the M2 phenotype [48, 49]. With its ability to muster M2 phenotype macrophages into the TME and increase metalloproteinase secretion to support metastases, the CSF-1-mediated pathway becomes an appealing therapeutic target for small molecule intervention [50].

### Extracellular matrix

**Fig. 2:**
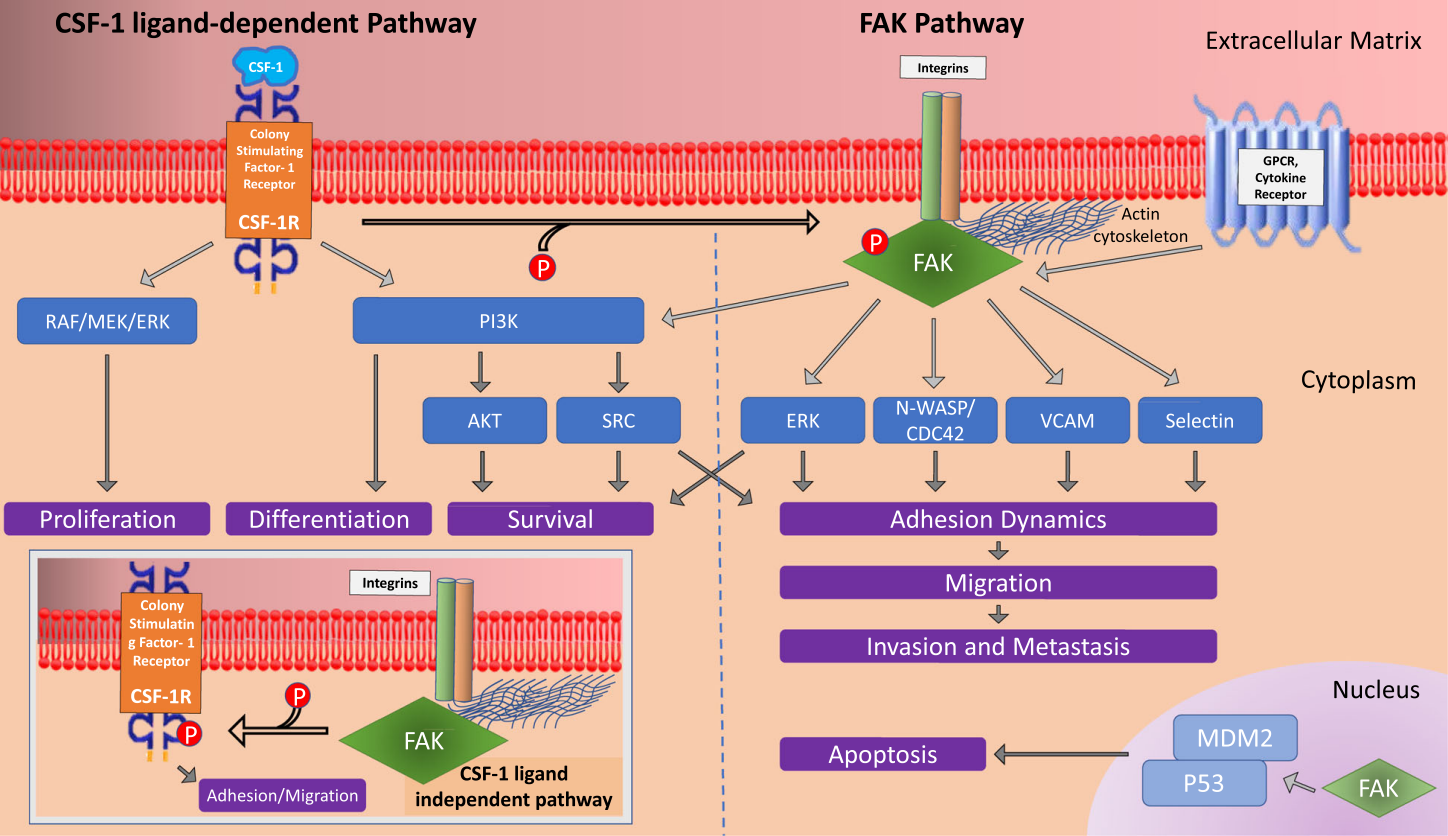
Signaling pathways for CSF-1 and FAK. CSF-1R predominantly modulates differentiation, proliferation and survival via PI3K or the RAF/MEK/ERK pathway. For the regulation of cell adhesion and migration, the binding of CSF-1 to CSF-1R leads to phosphorylation of FAK, which in turn activates numerous signaling pathways that lead to actin polymerization/cytoskeleton remodeling, adhesion dynamics and migration (via ERK, N-WASP/CDC42, VCAM and Selectin). However, like CSF-1/CSF-1R, FAK is also involved in cell survival via the PI3/AKT pathway. Interaction of ECM protein (e.g. fibronectin) with integrins can also activate FAK, which leads to ligand-independent phosphorylation of CSF-1R, and thus cell migration (inset on left lower portion of Fig. 1)

The tumor ECM functions more than a simple scaffold in which the cells and the lymphatic and vascular system reside; it also plays a critical role in supporting the inflammatory milieu needed for tumor progression and metastasis [51, 52]. The ECM is a depot for cytokines, growth factors and other molecules, and their effects are communicated via the integrins that couple the ECM to the actin cytoskeleton. Interactions between TAMs and ECM proteins can promote metastasis, and in this regard, CSF-1 and FAK serve as important examples of how the interaction between the ECM and the inflammatory milieu leads to cancer progression (Fig. 2) [52]. CSF-1 signaling via CSF-1R leads to increased FAK phosphorylation in macrophages, and FAK then mediates cell adhesion turnover. Without FAK, macrophages cannot form stable protrusions (i.e. broad lamellipodia), nor form a leading edge for migration [53, 54]. Thus, chemotaxis by macrophages to a chemo-attractants such as CSF-1 is precluded, as is random migration, leading to decreased macrophages at sites of inflammation. In addition, ECM protein fibronectin’s interaction with integrins activates FAK and leads to ligand-independent phosphorylation of CSF-1R and subsequent myeloid cell migration [55].

## Advantages of immunomodulatory small molecule inhibitors

In cancer, molecular targeting of cellular pathways typically utilizes two pharmacologic modalities, mABs or small molecule inhibitors in order to delay or overcome drug resistance. To overcome the lack of therapeutic response to checkpoint inhibition monotherapy, combination therapy of multiple immune checkpoint mABs have been attempted [56]. These combinations indeed increase the rate of response in patients, but not without the expected increase in the rate of toxicities, leading to dose reductions and even permanent discontinuation [57]. For example, in subjects with melanoma, the treatment combination of anti-PD-1 and anti-CTLA-4 blockade led to significantly more adverse events compared to anti-PD-1 monotherapy (55–60% vs. 10–20% high-grade), where nearly 80% of subjects treated with the combination therapy discontinued therapy as a result of toxicity [56, 57]. Combination therapies of monoclonal checkpoint inhibitors with immunomodulatory small molecule inhibitors, however, have been better tolerated, which can be attributed to the lower molecule weight of these small molecules affecting their more favorable pharmacokinetics, oral bioavailability and the lower degree of overlapping toxicities when administered in combination with checkpoint inhibitors, compared to mABs [58].

Anti-tumor small molecule inhibitors are generally much smaller than mABs, with small molecule inhibitors having a molecular weight of ≤500 Da (da, g/mol) or 500–1000 da, depending on the studies’ definitions, whereas mAbs are substantially larger with molecular weights on the order of many kilodaltons [59, 60]. These size differences have various implications, particularly for drug development, administration and cell penetration [20]. As oncologic drugs undergo translational investigation and development, they progress from target selection/validation to chemical hit, culminating with lead optimization to become an ideal candidate for clinical trials. In chemical hits that have similar potency, the one with a lower molecular weight is more advantageous and drug candidates with a molecular weight > 550 da have an increased likelihood of failing drug development [61]. It is thought that due to the difference in molecular size, small-molecule agents might be more efficient in tissue penetration, tumor retention and blood clearance compared to IgG subclasses of mABs [62]. Because of their large size, mABs cannot pass through the cell membrane and can only act on the cell surface or on secreted molecules. Small molecule inhibitors, on the other hand, can target molecules both intra- and extra-cellular by having the ability to pass into the cytoplasm [63]. This difference has particular implications on proteins and pathways which are non-receptor kinases, such as FAK. Additionally, kinases like FAK, which are implicated in tumor growth and metastasis, traffic to and are present in the nucleus, consequently effecting gene regulation in a kinase-independent manner (Fig. 2) [64]. Targeting of such proteins and functions, like nuclear FAK via mABs is not a viable option and as such highlights the importance of small molecular targeting. Furthermore, unlike mABs, small molecule inhibitors are far less expensive and require less complicated processes for development [65]. In general, the adverse effects associated with small-molecule inhibitors are mild, which are usually dermatologic or gastrointestinal in nature, as noted with epidermal growth factor receptor (EGFR) small molecule inhibitors for lung cancer or FAK inhibitors which are currently under investigation in numerous malignancies [62]. With regard to target specificity, small molecule inhibitors are generally considered to be less specific than mABs. Despite this, small molecular inhibitors can impact several signaling pathways at plasma concentrations that are clinically feasible [62].

Small molecule inhibitors have a multi-faceted effect on the TME. Multiple studies have shown that targeting a specific molecule in a cellular pathway not only affects the downstream actions of the pathway, but also has a cascading effect on a multitude of different mechanisms, including but not limited to epigenetic modification, T-cell activation and myeloid-derived immune suppression [66, 67]. For example, MEK inhibitors target the RAS-RAF-MEK1/2-ERK1/2 pathway, which is constitutively activated by KRAS mutations and renowned for its role in cellular proliferation and tumorigenesis, but MEK inhibitors have also been shown to increase T-cell infiltration and reduce MDSCs in the TME [68]. Other small molecule inhibitors, such as inhibitors of indoleamine-2,3-dioxygenase (IDO), have also been shown to affect a myriad of immune cell types [20, 69, 70]. These small molecules can overcome traditional checkpoint inhibitor resistance through non-redundant immune pathway mechanisms and are thus viable options for combination therapy with checkpoint inhibition. Over the last decade, numerous small molecule inhibitors with immunomodulatory properties have been developed, and are actively being investigated as therapeutic modalities.

## CSF1R inhibition

CSF-1R signaling is a critical modulator of the mononuclear phagocyte system and thus immunosuppression within the TME [71]. CSF-1R is a transmembrane, tyrosine kinase receptor which is activated by the binding of two ligands: CSF-1 and IL-34 [72]. Upon ligand attachment, receptor dimerization occurs, subsequently followed by the expression and phosphorylation of critical tyrosine residues in the intracellular environment [73, 74]. Consequently, an activating platform for numerous macrophage-related signaling cascades, including the PI3K/AKT, SRC, mitogen-activated protein kinase and FAK, is created (Fig. 2). These signaling cascades are intricately involved in the development, differentiation, propagation, survival and migration of TAMs and other myeloid cells [75–78]. The blockade of CSF-1/CSF-1R leads to the reduction of TAMs in the TME, reprogramming of TAMs to augment antigen presentation and reinforcement of T-cell activation within the TME. The downstream effects of CSF-1/CSF-1R blockade create an environment with decreased immune suppression and increased interferon response, impeding tumor growth [24].

### Preclinical evidence

PLX-3397 was one of the first small molecule inhibitors of the CSF-1 pathway, and not only is it a potent tyrosine kinase inhibitor of CSF-1R, but it also targets cKIT and FLT3. In preclinical lung adenocarcinoma mouse models, PLX-3397 was shown to modify TAM distribution in the TME and decrease tumor burden [79]. Similarly, in syngeneic mouse models of BRAF V600E-mutated melanoma, PLX-3397 combination therapy with adoptive cell transfer immunotherapy, showed reduction in TAMs and increase in tumor infiltrating lymphocytes leading to increased release of IFN-γ [80]. When combined with BRAF inhibitor, PLX4032, in similar melanoma mouse models, PLX-3397 was shown to substantially reduce M2 phenotype macrophage recruitment, leading to significant tumor growth suppression [81]. In this same study, expression of PD-1 and PD-L1 was increased on intratumoral CD11b + myeloid cells, suggesting an attenuating mechanism on the combination therapy of BRAF and CSF-1R inhibition. When PD-L1/PD-1 inhibitory therapy was added to PLX4032/PLX-3397-treated mice, outcomes improved. This suggested a role for PD-L1/PD-1 blockade as adjunctive therapy to PLX-3397.

In pancreatic cancer xenograft models, Zhu et al. demonstrated that CSF-1R blockade with PLX-3397 decreased CD206 TAMs (i.e. M2 phenotype macrophage) within the TME and reprogrammed the remaining TAMs towards an anti-tumor phenotype [24]. This study also reaffirmed that CSF-1/CSF-1R inhibition altered T-cell checkpoint signaling, as was previously shown in melanoma models treated with PLX-3397. Zhu et al. found that PD-1 and PD-L1 expression on TAMs and CTLA-4 expression on CD8+ T-cells were upregulated by CSF-1R inhibition. The addition of PD-1 or CTLA-4 antagonists in conjunction with PLX-3397 led to a more than 90% reduction in tumor progression. This study again suggested that small molecule inhibition with CSF-1R can enhance checkpoint blockade therapy.

Other small molecules targeting CSF-1R, such as BLZ945 and ARRY-382, have also been developed and have shown similar preclinical outcomes to PLX-3397. BLZ945 is a unique CSF-1R inhibitor with the ability to penetrate the central nervous system (CNS). For this reason, it was investigated in glioblastoma multiforme (GBM) mouse models [82]. Despite multiple tumor-specific factors in GBM that dampened TAM depletion, BLZ945 was found to reduce polarization towards an M2 macrophage phenotype [82]. BLZ945 ultimately inhibited tumor growth and led to increased survival in GBM [82]. CSF-1R inhibition and its anti-tumor effects are not limited to solid tumor subtypes, but have also been appreciated in hematologic malignancies, where CSF-1R expressing macrophages within the TME stimulate tumor survival. For instance, when two inhibitors of CSF-1R, GW-580 and ARRY-382, were added to the sera of chronic lymphocytic leukemia patients in vitro, it resulted in decreased tumor-supportive macrophages and depleted CD14+ monocytes in the TME [83].

Studies have also shown that CSF-1R inhibition may sensitize tumor cells to more traditional cytotoxic therapy [84]. In lung cancer preclinical models, CSF-1R inhibition has been shown to sensitize cisplatin-resistant lung cancer cell populations against platinum-based therapy, further supporting its roles as an adjunctive agent not only to immunotherapy but also chemotherapy [85].

### Clinical studies

Preclinical investigations of PLX-3397, BLZ945 and ARRY-382 have paved the way for clinical studies of CSF-1R inhibition via small molecules and mABs in diverse tumor types from GBM to pancreatic, ovarian and colorectal cancers (Table 1). Among these small molecule inhibitors of the CSF-1/CSF-1R pathway, PLX-3397 (Pexidartinib) currently has the most clinical data. PLX-3397 was evaluated in 37 patients with recurrent GBM, where it was tolerated well and with excellent CNS penetration. However it had minimal clinical efficacy, as only 8.6% had a progression free survival of 6 months, with no objective responses observed [86]. A phase I dose escalation study of PLX-3397, among multiple advanced tumor types (CRC, ovarian, breast, leiomyosarcoma, PDAC, lung) also noted a favorable safety profile and a marked reduction in a defined subset of circulating monocytes (CD14^dim^/CD16^+^) [87]. In these studies, the most common side effects noted for PLX-3397 were fatigue, nausea, anemia, decreased appetite, rash, hair depigmentation, headache, constipation and transaminitis. Most recently, a pivotal phase III study (ENLIVEN) evaluating PLX-3397 was completed in 120 patients with advanced symptomatic tenosynovial giant cell tumors (TGCT), also known as pigmented villonodular synovitis, a malignancy in which surgical tumor resection often results in worsening functional status and morbidity [88]. Overexpression of CSF-1 is associated with this rare tumor type and the disease itself is linked to significant reactive inflammation in the tumor environment, suggesting a role of CSF-1 targeted therapy [89]. ENLIVEN showed that PLX-3397 significantly reduced the tumor size with a 39% overall tumor response, compared to no tumor response in patients treated with placebo [88].

**Table 1:** Active Recruiting Current Clinical Trials with CSF-1R Inhibitors in Various Malignancies

NCI Identifier	Study Description	Tumor Type	Drug Combination with CSF-1R inhibitor*	Phase
NCT02777710	Evaluation of Safety and Activity of an Anti-PDL1 Antibody (DURVALUMAB) Combined With CSF-1R TKI (PEXIDARTINIB) in Patients With Metastatic/Advanced Pancreatic or Colorectal Cancers	Advanced Cancers, Colorectal, Pancreatic Cancer	Durvalumab Pexidartinib(PLX-3397)*	I
NCT02401815	PLX9486 as a Single Agent and in Combination With PLX3397 or PLX9486 With Sunitinib in Patients With Advanced Solid Tumors	Gastrointestinal Stromal Tumors	PLX-3397* PLX9486 Sunitinib	I/II
NCT02071940	PLX3397 KIT in Acral and mucosal Melanoma	Melanoma	PLX-3397*	II
NCT02584647	PLX3397 Plus Sirolimus in Unresectable Sarcoma and Malignant Peripheral Nerve Sheath Tumors	Sarcoma, Malignant Peripheral Nerve Sheath Tumors	Sirolimus PLX-3397*	I/II
NCT03069469	Study of DCC-3014 in Patients With Advanced Malignancies	Advanced Malignant	DCC-3014*	I
NCT02880371	A Study of ARRY-382 in Combination With Pembrolizumab for the Treatment of Patients With Advanced Solid Tumors	Advanced Solid Tumors, Platinum Resistant ovarian cancer, pancreatic ductal adenocarcinoma	ARRY-382* Pembrolizumab	I/II
NCT02829723	Phase I/II Study of BLZ945 Single Agent or BLZ945 in Combination With PDR001 in Advanced Solid Tumors	Advanced Solid Tumors	BLZ945* PDR001	I/II

*There are also clinical trials evaluating monoclonal Antibodies Targeting CSF-1R, including NCT02718911, NCT03238027, NCT02471716, NCT03101254, NCT03336216, NCT03431948, and NCT03335540, which are assessing LY3022855, SNDX-6352, FPA008 (Cabiralizumab), LY3022855, FPA008, FPA008 and FPA008, respectively

To enhance the clinical responses garnered by CSF-1R inhibition, numerous ongoing clinical trials are combining small molecules inhibitors or mABs of CSF-1R with immunotherapy and/or cytotoxic chemotherapy (Table 1). Recently, preliminary efficacy data from a phase 1 dose escalation and expansion trial by Wainberg et al. looking at a combination of anti-CSF-1R (cabiralizumab) and anti-PD-1 mABs reported an objective response rate of 13% (four patients) amongst a cohort of 31 patients with advanced pancreatic cancer and most of whom were heavily-pretreated. All four of these patients had microsatellite stable disease, which historically has been unresponsive to PD-1/PD-L1 blockade. Three of these patients experienced a partial response and one had stable disease, with two patients experiencing a reduction in target lesions of 50% or more [90]. Despite cabiralizumab being a mAb, this study provides evidence to support further investigation of small molecules targeting CSF-1R in combination with immunotherapy. Small molecule inhibition of CSF-1R with chemotherapy has also shown promising clinical results. For instance, ABT-869, another novel small molecule inhibitor of CSF-1R, in combination with weekly paclitaxel in a small phase I study, showed clinical activity in 2 of 5 patients [91].

In conclusion, preclinical and clinical studies have demonstrated the benefit of combining CSF-1R inhibitors with immunotherapy and/or chemotherapy. This is an active area of research where CSF-1R inhibitors are a novel class of immunomodulatory therapeutics that have the capacity to unlock the full potential of immunotherapy in advanced malignancies.

## FAK inhibition

FAK is a nonreceptor protein tyrosine kinase that is often upregulated in many malignancies, and is downstream to the signaling of integrins and growth factor receptors that maintain the neoplastic nature and survival of cancer cells (Fig. 2). Also through cancer stem cell (CSC) renewal, it controls a wide range of integral cellular functions [92, 93]. Additionally, FAK activation, via autophosphorylation at Tyrosine-397, increases with tumor progression [94]. Activated FAK mediates a multitude of cellular and extracellular process involved in cell invasion and metastases, from cell attachment to the ECM, remodeling, focal adhesion formation and turnover, as well as expression of matrix metalloproteinases [95] (Fig. 2).

### Preclinical evidence

As such, the effect of FAK is not only limited to cells of tumor origin, but also to cells within or recruited to the TME. FAK signaling is intimately involved in various aspects of the TME, particularly immunosuppression and stromal alterations. Studies have shown that inhibition of FAK diminishes the recruitment and migration of CAFs [96]. CAFs are abundant in the tumor stromal environment and are implicated in tumor growth, angiogenesis, metastasis and drug resistance [97]. In pancreatic cancer, the stroma and TME are characterized by increased collagen deposition with an elevated fibrotic response and infiltration of CAFs [98]. In a study by Stokes et al., pancreatic tumors from animals treated with PF-562,271 (VS-6063, [defactinib] a small molecule inhibitor of FAK) led to a significant decrease in the number CAFs and a significant decrease in tumor cell proliferation [96]. Additionally, CAFs have been shown to suppress CD8+ T-cells, where those cells conditioned by CAFs had diminished cytotoxic capacity. Furthermore, CAFs are associated with T-cell dysfunction via PD-L2 and fas ligand engagement [99].

Beyond CAFs, many preclinical studies have revealed that FAK signaling is closely involved in the activity of MDSCs, TAMs and Tregs within the TME [64, 67]. In squamous cell carcinoma mouse models, small molecule FAK inhibitor, VS4718, was shown to decrease immunosuppressive MDSCs, TAMs and Tregs, which then led to increased CD8+ T-cells within the tumor and enhancement of CD8+ T-cell-mediated suppression of cancerous cells [66].

In many tumors, particularly pancreatic cancer, studies have shown that the efficacy of traditional cytotoxic chemotherapy and immunotherapy can be improved by decreasing the density of peri-tumor stroma and the infiltration of myeloid cells [100, 101]. Jiang et al. demonstrated that FAK inhibition can reduce both fibrosis and immune-inhibitory myeloid cells [67]. Using genetically modified KPC (p48-Cre/LSL-Kras^G12D^/p53^Flox/Flox^) mouse models, Jiang et al. found that FAK inhibitor, VS-4718, decreased the stromal density of the pancreatic tumors, and reduced MDSCs, TAMs and Tregs infiltration into the tumor. They also discovered that FAK inhibition potentiated anti-PD1 therapy, thereby decreasing tumor burden and improving survival. Mice treated with gemcitabine, anti-PD-1 therapy and FAK inhibition had a 2.5-fold increase in median survival compared to those treated without FAK inhibition. Tumors from mice treated with FAK inhibition, gemcitabine and anti-PD1 therapy also had a significantly increased number of tumor-infiltrating CD8+ T-cells compared to mice treated with gemcitabine and anti-PD1 therapy without FAK inhibition [67].

An additional benefit of FAK inhibition is its ability to decrease CSCs. CSCs are unique cells within a tumor that are capable of self-renewal, able to generate more cancer cells with heterogeneous differentiation and typically resistant to standard therapies, leading to tumor resistance, recurrence and metastasis [102, 103]. In preclinical malignant mesothelioma models, standard cytotoxic therapies such as pemetrexed, cisplatin, gemcitabine and vinorelbine have been shown to increase CSCs, but when FAK inhibition is added, CSCs decrease [104]. CSCs do not exist in isolation, but are influenced by critical factors within the TME such as cytokines, small RNAs, TAMs and fibroblasts, which impact their unique niche [105, 106]. These factors regulate the invasiveness, metastatic potential and differentiation of CSCs, as well as confer a tumor-protective phenotype.

### Clinical studies

Based on these promising preclinical studies elucidating the role of FAK inhibition in modulating the immune milieu and fibrosis within the TME, clinical trials are investigating combination therapy of FAK inhibitors with cytotoxic chemotherapy and/or immunotherapy (Table 2). FAK overexpression has been noted in many tumor types, with associated negative prognostic factors, including HCC, NSCLC, colon, breast, pancreatic and ovarian cancers [26]. One study found that 68% of invasive ovarian cancers overexpressed FAK, which was associated with significantly higher tumor stages and tumor grades, positive lymph nodes and distant metastasis, and supported investigation of FAK inhibitor in advanced ovarian cancer [107].

**Table 2:** Active Recruiting Current Clinical Trials with FAK Inhibitors in Various Malignancies

NCI Identifier	Study Description	Tumor Type	Drug Combination with FAK inhibitor*	Phase
NCT03287271	ROCKIF Trial: Re-sensitization of Carboplatin-resistant Ovarian Cancer With Kinase Inhibition of FAK	Ovarian	Paclitaxel, Carboplatin VS-6063 (defactinib)*	I/II
NCT02758587	Study of FAK (Defactinib) and PD-1 (Pembrolizumab) Inhibition in Advanced Solid Malignancies (FAK-PD1)	NSCLC, Mesothelioma, Pancreatic Neoplasms	Pembrolizumab Defactinib*	I/II
NCT02523014	Vismodegib and FAK Inhibitor GSK2256098 in Treating Patients With Progressive Meningiomas	Meningioma	Vismodegib GSK2256098*	II
NCT02546531	Defactinib Combined With Pembrolizumab and Gemcitabine in Patients With Advanced Cancer	Advanced solid tumors, Pancreatic Cancer	Gemcitabine, Pembrolizumab Defactinib	I
NCT02695550	Study of Safety, Efficacy and Pharmacokinetics of CT-707 in Patients With ALK-positive Non-small Cell Lung Cancer	NSCLC	CT-707*	I
NCT03727880	Study of Pembrolizumab With or Without Defactinib Following Chemotherapy as a Neoadjuvant and Adjuvant Treatment for Resectable Pancreatic Ductal Adenocarcinoma	PDAC	Pembrolizumab Defactinib	II

Preliminary data from a phase 1 dose escalation study of Defactinib, anti-PD1 therapy pembrolizumab and gemcitabine in patients with advanced solid tumors, with an expansion cohort for patients with advanced PDAC, have already shown that the combination therapy is well-tolerated (NCT02546531) [19]. Defactinib (VS-6063) is a selective adenosine triphosphate (ATP) that is a competitive and reversible inhibitor of human FAK and one of many FAK inhibitors in development. In addition, the study also reported that biopsies in patients with PDAC have decreased p-FAK and changes in T-cells infiltration following treatment [19]. The most common side effects noted with FAK inhibition were nausea, vomiting, pruritus, fevers and myalgias. The expansion cohort is currently ongoing with pending correlative and efficacy data. This phase I study and preclinical work with FAK have led to a phase II clinical trial (NCT03727880) combining neoadjuvant and adjuvant pembrolizumab and defactinib following neoadjuvant standard of care chemotherapy in subjects with high-risk resectable PDAC. This study will evaluate if reprograming the TME following chemotherapy by modulating TAMs and MDSCs with FAK inhibition can potentiate anti-PD-1 antibody therapy, and thus lead to improved effector T-cell infiltration and pathologic response.

Defactinib was also studied in malignant pleural mesothelioma in a phase II study with 30 participants. Objective partial response was observed in 13%, stable disease in 67% and progression in 17% of patients. This study also investigated the biological and immune implications of FAK inhibitor therapy on the TME, and showed that treatment with defactinib in malignant pleural mesothelioma resulted in a 75% reduction in p-FAK. Within the TME of treated subjects, there was increased naïve CD4+ and CD8+ T-cells, reduction of myeloid and Treg immuno-suppressive cells and reduction of exhausted T-cells and peripheral MDSCs. This study showed that defactintib has both therapeutic and immunomodulatory effects in patients with an aggressive malignancy, such as malignant pleural mesothelioma [108]. Currently there is a dose escalation study is underway in Europe, where defactinib is being combined with pembrolizumab in refractory advanced solid tumors and expansion cohorts in NSCLC, mesothelioma and pancreatic neoplasms (NCT02758587).

Defactinib has also shown clinical promise in combination with chemotherapy. Based on evidence showing elevated FAK expression in ovarian cancer, defactinib has also been studied in 18 patients with advanced ovarian cancer in combination with weekly paclitaxel, where a decrease of p-FAK was observed in all 3 patients who underwent paired biopsies. One patient had a complete response by RECIST, one patient an ongoing partial response of > 6 months and one patient with ongoing stable disease of > 8 months [109].

FAK has tremendous potential as a small molecular target, as it is implicated in modulating the immunosuppressive components of the TME, as well as the resistant and aggressive phenotype of CSCs. FAK inhibition leads to anti-tumor activity and when used in combination therapy, has the potential to increase the effectiveness of traditional cytotoxic chemotherapy and immunotherapy, particularly for aggressive and refractory malignancies.

## Conclusion

Until recently, cytotoxic chemotherapy, surgery, radiotherapy and targeted therapy were the pillars of cancer treatment. Immunotherapy has now become the fifth pillar of oncologic care, but its rise to prominence has not been without failure. Despite the success of checkpoint inhibition, numerous obstacles remain to unlocking the full potential of immunotherapy. The TME is a reservoir of these obstacles, and these obstacles tip the scales towards the immune escape of tumors. However, the TME also provides rational targets for small molecule inhibition through which immunomodulation can occur. Contemporary studies as outlined in this review suggest that small molecule immunomodulatory inhibitors, in conjunction with immunotherapy, may be able to overcome these obstacles within the TME and revert the immune system to a more anti-tumor state. Further research into the TME, small immune-modulating molecular targets and cancer immunology will hopefully realize the full potential of combination therapy with checkpoint inhibition and in turn provide clinically meaningful outcomes beyond what we have experienced in the modern era with traditional cytotoxic chemotherapy, radiotherapy, targeted therapy and immunotherapy. High quality correlative studies in parallel with clinical trials will be essential to unravel the mechanisms behind combination therapy.

## Data Availability

Not applicable.

## Abbreviations

CAF: Carcinoma-associated fibroblasts
CNS: Central nervous system
CRC: Colorectal cancer
CSC: Cancer stem cells
CSF-1: Colony stimulating factor-1
CSF-1R: Colony stimulating factor-1 receptor
CTLA-4: Cytotoxic T-lymphocyte associated protein-4
ECM: Extracellular Matrix
EGFR: Epidermal growth factor receptor
FAK: Focal adhesion kinase
HCC: Hepatocellular carcinoma
IDO: Indoleamine-2, 3-dioxygenase
IFN-γ: Interferon gamma
mABs: Monoclonal antibodies
MDSC: Myeloid-derived suppressor cell
NSCLC: Non-small cell lung cancer
PD-1: Programmed cell death protein-1
PDAC: Pancreatic Ductal Adenocarcinoma
PD-L1: Programmed cell death protein ligand-1
p-FAK: Phosphorylated FAK
TAM: Tumor-associated macrophage
TGCT: Tenosynovial giant cell tumors
TME: Tumor microenvironment
TNF-α: Tumor necrosis factor-alpha
Treg: Regulatory T-cell
